# The Origin, Progress and Present Condition of Dental Science

**Published:** 1879-06

**Authors:** Geo. H. Chewning


### ARTICLE II.

### *The Origin, Progress and Present Condition of Dental Science.*

BY GEO. H. CHEWNING, D. D. S.

From an Essay read before the Virginia State Dental Association.

The Dental Art has generally been considered of modern date; it is however of a much more ancient origin than is usually supposed.

In Egypt we have the first mention of the dental art as a distinct branch of the medical profession.

It is stated by Herodotus, the Grecian historian, that the art of medicine is so practised in Egypt that there is found an individual healer for each individual disease. Hence the whole country was filled with healers, some to take charge of disorders of eyes, others of the head, others of those of the teeth, and others of secret disease, &c.

Now as these several branches were hereditary, it might have been presumed that the professors of them would in process of time attain great perfection each in his own branch, and accordingly the skill of the medical men of Egypt was long the wonder and admiration of the world, and the monarchs of Persia and other countries, for many ages, employed Egyptians alone as their physicians and surgeons, till the surgery of the Greek School at length surplanted them.

During the time of Hippocrates, the first good observer, and the first practical physician whom the world knew, and who furnishes in philosophy one of the first examples of that analytical method of cultivating science, which is commonly considered proper to modern times, and of which ancient times furnish but few instances, one of his practices in pursuit of study of anatomy was to visit frequently the burial grounds of the cities in which he resided, and his description, therefore, of bones and teeth are not only the best which had hitherto appeared, but the best part of his anatomical writings. He describes in various parts of his work as well the functions and periods of appearance of the several teeth, as their principle diseases, and plan of treating them both by manual operations and by dentifrices

In Celsus we meet for the first time, anything like explicit directions in regard to extraction of teeth. Such as the necessity of severing the tooth from the alveolar process before attempting to remove it, also the description of the frames of the instruments; how scarifying the gums, and of stopping carious teeth with lead and other substances, though the latter practice seems to have been more to prevent their breaking during the operation of extracting.

One of the forceps described like the bill of the parrott —very much like the hawk's bill forceps, of the present day.

From the poet Martial, who flourished about that time, we find the first mention of *false teeth* made of bone or ivory, and mentioned more specially the Roman Ladies, as resorting to that means, to increase their personal attractions.

We now come to an era in the history of medicine, which ranks next to that of Hippocrates. I mean the era of

"Claudius Galen." About the time of his appearance, the profession was split into all kinds of sects, the Methodic, Pneumatic, Empiric and many others, but Galen appeared and all hid their diminutive heads, like the stars at the rising of the sun. He was born at Pergarnus, about 130 years after the Christian era, and studied philosophy and medicine successively at Smyrna, Corinth and Alexandria. At the age of 34 he settled in Rome, and remained there with only temporary visits to other places till his death. Galen was obviously a man of wonderful acuteness and industry, and left behind him many medical writings.

His description of the practice of extracting teeth, and dentifrices and washes, were about the same as others that preceded him.

For some centuries after the time of Galen the progress of medical science retrograded the decline and division of the Roman Empire, which happened about 400 years after Christ, having entailed upon science and literature, such discouragement as was incompatible with their successful cultivations. Towards the middle of the seventh century the few remains of learning that were left, were almost extinguished by the barbarous and bigotted Saracens, who in 640 made themselves masters of Alexandria, which had contained for nearly a thousand years the greatest school for learning, together with the most valuable library in the world at that time. This the savage disciples of the ignorant Mahomet entirely destroyed, assigning as their reason, "that if the books contained only what was in the Koran, they were useless, and if more they were pernicious." We may form some idea of the extent of their destruction, when we learn that the books so condemned were sufficient to furnish with fuel for more than six months, no fewer than four thousand hot baths.

The Saracens, however, were not designed to continue savages, for even their Califs were soon made sensible of the advantage of science and learning, and began 100 years after the destruction of the Alexandria library to give large premiums for the translation into Syrica and Arabic, of such Greek writers as they could procure.

About the eleventh century we find from the works of Albucasis, the prince of Surgeons, a much more general description of Dentistry. First the extraction, then the material for filling, and how it should be introduced, with a description of the file; also describing no less than twelve instruments for cleansing the teeth of Saliva Calculus.

He was acquainted also with the use of false teeth, describing them as made of bone, &c., and being retained in their places by gold wire as were loosened or transplanted natural teeth.

The first time we meet with the appellation of Surgeon Dentist was to Gillies, in 1622, in France, to undergo a regular examination, and it is from this period perhaps, that we may date the establishment of the dental art, as a distinct branch of the medical practice.

In the latter part of the sixteenth century, but more particularly in the beginning of the seventeenth century began to assume a different and more interesting aspect, so much so at least as to have engaged the attention of many eminent writers and naturalists, and particularly some whose pursuits have been directed to the treatment of the diseases of the mouth and teeth. Among these were Laudemey, of Paris, who from the knowledge he had acquired and the celebrity he enjoyed, was sent for to the Court of Spain, in the year 1716, to perform an operation upon the mouth of Philip, then Sovereign of the Kingdom. We are not aware of his ever writing anything upon the subject of his profession, but we find that in 1723 he was acting in the capacity of Dental Surgeon to his catholic majesty Philip, King of Spain.

At that period we also find that Fauchard, a Dental Surgeon, of Paris, was engaged in writing a work on the subject, and which was published in 1728 in two volumes, and in which was made the first attempt to systemize and treat methodically the dental art as a distinct and separate branch of the Medical Science; hence he has ever since been considered, and justly styled the Father of Dentistry.

He was one of the first to offer any satisfactory directions for obviating defects in the palate, and gives plates of five kinds of obturators for that purpose.

From the year 1728 to the year 1806, not less than 7 or 8 dental works appeared. About the time that Berdmore wrote his practical treatise, (which was in the year 1770,) Mr. Hunter was engaged in his work, on the Natural History of the Teeth. From that period to 1798, the year in which Mr. Blake wrote, no other work appeared that we know of. Mr. Fox contributed his share of that kind of knowledge, so essentially important and necessary to assist those who were practically engaged in operations so intimately connected with the health and comfort of thousands.

And last, though by no means least in importance, the work published by Mr. Thomas Bell, which well fulfilled the purpose he designed, I doubt not at that time, was equal to any that had been written on Dental Surgery in the world.

Having thus given a brief sketch of the origin of Dentistry in the old world, will now try and direct your attention to the new world.

The first opportunity offered to any in America to obtain a knowledge of the profession was through a friend, dentist by the name of Lee Marie, who offered his services to the public, during the revolutionary war. He had probably acquired a knowledge of the profession in his own country, where it had long been cultivated, and not without some pretentions to skill in practical operations, especially in the transplanting of teeth from the mouth of one person to that of another, which he frequently performed. He likewise undertook to instruct some three or four in the profession. About the same time we have mention of two gentlemen who had probably acquired the knowledge of their profession in England,—Whitelock and Hoofindale.

Mr. John Greenwood was the first Native American Dentist, and was endowed with no ordinary share of ingenuity and mechanical tact. He commenced his professional course in the city of New York, about the year 1789 or 90, at the latter part of which period we believe he stood alone in his profession, enjoying almost the exclusive patronage and confidence, not only of the inhabitants of that city, but of the father of his Country, George Washington himself, for whom he made an entire set of teeth, (which was on exhibition at the Centennial, and doubtless examined and admired by many of our fraternity,) and is now in the museum of the Baltimore College.

From the time of Greenwood, the advances of valuable improvements in practical dental operations were onward and rapid, and that too by unlooked for and valuable auxiliaries. For about this time the importance and respectability of the profession was considerably enhanced by the auspices of the well known talent and ability of the elder Mr. Gardet, who having obtained a valuable fund of professional knowledge before leaving his native country, France, came and settled in Philadelphia, where he soon received the most flattering encouragement for his services.

About the same time the profession experienced another no less valuable or important accession in professional skill and talent, in the arrival of Dr. Edward Hudson, from Dublin, who likewise prefered Philadelphia for his future home, and there practiced for many years.

Thus while the profession was dispensing its benefits and usefulness to the Northward and Eastward, through the diligent and successful practice of Messrs. Greenwood and Woofendale and some few others, the profession was advancing with rapid strides to usefulness and respectability, by the skill and ingenuity of Messrs. Gardet and Hudson, of Philadelphia, who were dispensing the benefits and examples of their superior skill and professional talent to the West and South, where they were at all times considered as worthy of imitation, and ever sought after with that view.

History of American Dentistry says of Dr. Hayden. Horace H. Hayden was born the 13th of October, 1768: and at the age of fourteen went to sea as a cabin boy, voyaging to the "West Indies;" two years later, being thrown upon his own resources by the poverty of his parents he became apprenticed to an architect; which business he followed until his 24th year, when being in New York, and having occasion for the professional aid of a dentist, he visited Mr. Greenwood, and while under his treatment, determined to become a dentist, and procuring the few dental books that were then accessible, and not apprehending any deficiency in mechanical skill, he directed his course Southward in quest of a location, (Harris, D. D.,) and arrived in Baltimore in 1804, where he determined to remain. Dr. Hayden conceived the idea that the dental profession was capable of a much higher standard of scientific attainment; accordingly he began the study of general medicine, while continuing to operate as a dentist, and with such effect, that later in his life he received honorary degrees from the University of Maryland, and the Jefferson College of Philadelphia. In 1839 he was one of the petitioners to the Legislature of Maryland, to establish a Dental College, the Faculty to consist of Dental and Medical practitioners. After the establishment of the Institution, he at the age of seventy assumed the duties of its chair of Dental Physiology and Pathology. Dr. Hayden was also one of the founders of the American Society of Dental Surgeons, and was in 1840 elected the first President, which office he held to the time of his death, January 26th, 1844.

Dr. Thomas E. Bond, was also a member of the Faculty of the Baltimore College, from the time of its foundation till his death, which occurred a few years since. He was the author of "Dental Medicine," which is one of the textbooks of the College. The American Journal of Dental Science, in speaking of this work says—The work of Professor Bond opens a new era in the history of Dental Surgery, pointing out as it does the pathological relationship between diseased teeth and other parts of the body. "It demonstrates in the fullest and most conclusive manner, the importance and absolute necessity of a thorough knowledge of Anatomy, Physiology, Pathology and Therapeutics, to the dentist." He also contributed largely to the literature of the day, and in conclusion, he was a great, a *good* man.

Professor Baxley held the chair of special Anatomy and Physiology for one course, resigning about three weeks previous to the beginning of the second session. "Professor Baxley as a lecturer and teacher of Anatomy ranked among the first in the country. His resignation did not proceed from any want of interest or confidence in the success of the College, but from the imperative demands upon his time, resulting from arduous professional duties."

Professor Chapin A. Harris was the founder of the Baltimore College of Dental Surgery. He was the first to consult with Prof. Hayden, in regard to establishing a college. He wrote the first "Practical Treatise on Dental Surgery," in 1837. Dr. Arthur says of this book: This is the first edition of a work which is now so well known, that any abstract of its contents would be a work of supererogation." It was the first entirely original work published in the country, for the use of the profession exclusively, and still in (1851) stands alone." In 1845 a second edition, very much enlarged and thoroughly revised, appeared under the title of "The Principles and Practice of Dental Surgery." Dr. Arthur also says of it: The book in this form was generally acknowledged to be the *best* practical treatise on Dental Surgery that had ever appeared in any language. This work having been revised by Profs. Austen and Gorgas, has since 1840 retained its place as a text book in most of the Dental Colleges.

In 1849 his Dental Dictionary appeared, since twice revised and enlarged by Prof. Gorgas, and not only are all Medical and Surgical terms explained with clearness, but we have also a collection of valuable knowledge that at that time could not be found any where else. Prof. Harris opened many new sources of Dental Science, and by his ability, by his judgment, and by his practice, enlarged the bounds of his art, and gave stability to his precepts, to which no one of us can have recourse without feeling personal obligation and unfeigned servience. He was a man of long and extensive experience, of originality of thought, talent, and genius, and under such impressions would I consider every memorial of his indefatigable mind, and every result of his curious and important investigations. He has and will forever have a bright and honored name in the profession of his choice. He was the head and corner-stone of the Baltimore College of Dental Surgery, and felt great anxiety and responsibility for its success while living, and his last aspirations probably were that those principles might be adopted and carried out which would perpetuate it.

Until the Baltimore College of Dental Surgery was established in 1839, no institute existed expressly for the purpose of giving instruction in Dentistry; up to that time instruction was exclusively confined to private tuition. For some time the scheme of establishing a College expressly for this purpose was regarded by the community as by many of the profession, as a difficult and doubtful undertaking, but thanks to the untiring exertions of its enterprising founder Chapin A. Harris, the enlightened policy of the State legislature, and to the cheering countenance and liberal support of the citizens of Baltimore, the experiment has been fully and successfully tried; the practicability and the great public utility of such an organization has been fully proven.

More than 800 students have received Diplomas from that institution; and there are but few if any of the Colleges that some of their faculty will not be found to be graduates of this College—also many of the Court dentists in foreign countries.

Where can there be found an Institute that has sent out better men or more skillful practitioners than the Baltimore College of Dental Surgery?

Let us glance at a few—Harris and his works, Bond, Arthur, Austin, Gorgas, Parmley and Hodgkin and a host of others celebrated for their writings, and some for their inventions. As time fails us however, we will only mention Dr. B. M. Wilkerson, as one of the inventors, who invented the "Wilkerson Chair" and Fountain Spittoon which you have all had or will have the pleasure of examining.

In 1845 the Ohio College was chartered, most of the faculty being graduates of the Baltimore College.

We have now in existence thirteen Colleges, and as nearly as we can learn over 2300 persons have received the degree of Doctor of Dental Surgery.

In 1840 the first Society was formed in the city of New York, called the "American Society of Dental Surgeons."

The next organization of the kind was the Virginia Society, the *first State* Society that was formed. It was organized the 12th of December, 1842, in Richmond. It was the first incorporated Dental Society in the world. Since that time we have had over 90 Associations, and at this present time they will average one for each State in the Union.

I should liked to have called your attention to the several discoveries made by dentists, such as Sulph. Ether, the application of Nitrous Oxide and many others, but time again fails me.

